# Study on the safety evaluation of latent tuberculosis treatment in high‑risk groups for tuberculosis development: Study protocol for a multi‑center prospective observational cohort study in Korea (STEP-TB)

**DOI:** 10.1371/journal.pone.0350186

**Published:** 2026-06-09

**Authors:** Yunkyeong Hwang, Yeonhee Park, Hyung Woo Kim, Seo Yun Jeong, Yoolwon Jeong, Helen R. Stagg, Ju Sang Kim, Jinsoo Min

**Affiliations:** 1 Division of Nephrology, Department of Internal Medicine, Daejeon St. Mary’s Hospital, College of Medicine, The Catholic University of Korea, Seoul, Republic of Korea; 2 Division of Pulmonary and Critical Care Medicine, Department of Internal Medicine, Daejeon St. Mary’s Hospital, College of Medicine, The Catholic University of Korea, Seoul, Republic of Korea; 3 Division of Pulmonary and Critical Care Medicine, Department of Internal Medicine, Incheon St. Mary’s Hospital, College of Medicine, The Catholic University of Korea, Seoul, Republic of Korea; 4 Department of Preventive Medicine, College of Medicine, Ewha Womans University, Seoul, Republic of Korea; 5 Department of Infectious Disease Epidemiology, London School of Hygiene & Tropical Medicine, London, United Kingdom; 6 Division of Pulmonary and Critical Care Medicine, Department of Internal Medicine, Seoul St. Mary’s Hospital, College of Medicine, The Catholic University of Korea, Seoul, Republic of Korea; Aurum Institute, SOUTH AFRICA

## Abstract

**Background:**

Tuberculosis preventive treatment (TPT) is essential for tuberculosis elimination; however, evidence on its safety and feasibility in medically complex, high-risk populations is limited. Concerns regarding adverse events frequently hinder treatment initiation and completion in routine clinical practice.

**Objectives:**

The Safety of Preventive Treatment in People at Risk for Tuberculosis (STEP-TB) study aims to generate real-world evidence on the safety of TPT among individuals at high risk of developing active tuberculosis disease and to identify factors associated with adverse events, treatment initiation, adherence, and completion.

**Methods:**

STEP-TB is a multicenter, prospective observational cohort study conducted at four university-affiliated hospitals in the Republic of Korea. Adults aged ≥19 years who are eligible for latent tuberculosis infection (LTBI) testing or TPT according to national guidelines will be enrolled, including individuals with chronic kidney disease, chronic lung disease, diabetes mellitus, immunosuppressive conditions, malignancy, or occupational risk. LTBI testing will be performed using interferon-gamma release assays, and TPT regimens will follow national guidelines. Participants initiating TPT will be followed for up to 12 months from treatment initiation. Those with negative LTBI results or without TPT will be also followed for up to 12 months. Adverse events, treatment adherence, and completion will be systematically assessed. Blood samples, including volumetric absorptive microsampling, will be collected in a subset of participants for pharmacokinetic and pharmacogenetic analyses.

**Outcomes:**

The primary outcome is the occurrence of adverse events during TPT. Secondary outcomes include TPT completion rates, predictors of non-initiation and discontinuation, and progression to active TB.

**Conclusion:**

STEP-TB will provide condition-specific, real-world evidence on TPT safety and implementation, informing clinical decision-making, patient-centered care, and national TB control policies to support the safe expansion of LTBI treatment strategies in Korea.

**CRIS Registration Number:** KCT0011063

## Introduction

Globally, tuberculosis (TB) is the leading cause of death among infectious diseases. Diabetes, human immunodeficiency virus infection, and malnutrition are recognized as major risk factors for developing active TB. According to the World Health Organization (WHO), achieving TB elimination by 2050 requires not only controlling active TB but also implementing treatment for latent TB infection (LTBI) [3]. LTBI is defined as infection with *Mycobacterium tuberculosis* in which a small number of viable bacilli persist in the host without being expelled externally; therefore, there is no transmission to others. Individuals are asymptomatic and have normal acid‑fast bacilli testing and chest radiography. Because residual bacilli cannot be directly detected, LTBI is diagnosed indirectly by measuring the host immune response to TB antigens. Individuals with LTBI are at risk of progressing to active TB disease, and multiple risk factors influence disease progression. Treating LTBI prevents progression to active TB at the individual level and reduces community transmission and overall TB prevalence [4,5].

According to the national TB surveillance data in the Republic of Korea, a total of 19,540 TB cases were notified in 2023, corresponding to an incidence rate of 38.2 per 100,000 population, representing a 9.2% decrease compared with 2022 [1]. Despite this continuous decline, the national TB burden in Korea remains disproportionately high relative to its socioeconomic status [2]. National policies for LTBI management are founded on cost‑effectiveness analyses, for which robust cohort data are essential. Korea has launched the “TB‑Free Korea” program to achieve TB elimination by 2030, with the goal of minimizing progression to active TB among persons with LTBI. To systematically implement LTBI policies, it is urgent to generate scientific evidence on the effectiveness and safety of TB preventive treatment (TPT). Furthermore, the factors associated with progression to active TB among individuals with positive LTBI testing have not been fully defined domestically.

Building a prospective cohort to collect clinical information on LTBI‑positive individuals is needed to establish a research platform for answering these questions. In this manuscript, we provide an overview of the design, methods and scope of this cohort study. This Safety of Preventive Treatment in People at Risk for Tuberculosis (STEP-TB) study is supported by the Korea National Institute of Health as a national research project. The study findings and datasets will be used as scientific evidence for developing the national TB control master plan in accordance with the TB Prevention Act. We aim to provide evidence to inform national TB policy by evaluating the safety of TPT in the high‑risk groups for active TB disease.

Among the medical high-risk groups for TB, including individuals with chronic kidney disease (CKD), chronic lung disease, diabetes mellitus, malignancy, immunosuppressive conditions, and occupational exposure, the safety of TB preventive treatment remains an important clinical concern. CKD is of special interest because patients with CKD have an increased risk of TB and may be more vulnerable to drug-related adverse events. This study aims to evaluate the safety of TPT in individuals at high risk of developing active TB disease in Korea using a multicenter prospective cohort design. The primary objective is to specifically investigate whether CKD is associated with an increased risk of adverse events during TPT. The secondary objective is to assess the association between CKD and TPT completion, to describe the TPT cascade of care among individuals at high risk of TB, and to evaluate the preventive effectiveness of TPT in reducing progression to active TB during the follow-up period. These objectives will allow a comprehensive understanding of both the safety and effectiveness of TPT in high-risk populations.

## Materials and methods

### Study design, and setting

The STEP-TB is a multi-center prospective observational cohort study conducted at four university-affiliated hospitals participating in the national Public-Private Mix Tuberculosis Control Project [3] in the Republic of Korea. The participating institutions include Seoul St. Mary’s Hospital, Incheon St. Mary’s Hospital, Yeouido St. Mary’s Hospital, and Daejeon St. Mary’s Hospital, all of which are tertiary-care centers affiliated with the Catholic University of Korea. Additional research sites may be added after protocol publication to expand recruitment capacity. Participant enrollment is scheduled to begin in October 2025 and will continue through December 31, 2027. Recruitment is expected to be completed by August 2027. Data collection is anticipated to conclude by September 2028. Data analysis is planned for October through December 2028, and the reporting of study results is expected to be completed in January 2029.

### Study population

The source population comprises individuals who are undergoing, scheduled to undergo, or have previously undergone latent TB infection (LTBI) testing at the participating hospitals. Eligible participants include those who (1) are scheduled for LTBI testing, (2) have already completed LTBI testing, (3) are currently receiving TB preventive treatment, or (4) have completed TPT within the past two years.

The inclusion and exclusion criteria are summarized in Table 1. The study targets adults aged 19 years or older, encompassing all individuals eligible for LTBI testing and treatment as defined in the “Korean Guidelines for Tuberculosis (fifth edition, 2024)” [4] and the “National Tuberculosis Control Manual (2025)”. This also includes employees working in congregate settings who are mandated to undergo LTBI screening under the TB Prevention Act [5]. Individuals with a history of treatment for active TB disease are excluded from participation.

**Table 1:** Eligibility criteria for participation in the STEP-TB cohort.

Category	Criteria
Inclusion criteria	1. Aged ≥ 19 years.
	2. Individuals eligible for LTBI testing or treatment who meet one or more of the following conditions: • Contact of a patient with infectious TB. • Person living with HIV. • Receiving or scheduled to receive immunosuppressive therapy. • Planned treatment with TNF antagonists, other biologic agents, or small-molecule inhibitors. • Presence of fibrotic or calcified lesions suggestive of spontaneously healed TB on chest radiograph. • Receiving or scheduled to receive prolonged corticosteroid therapy. • Having autoimmune disease. • Having chronic kidney disease, including those on dialysis. • Having hematologic malignancy or solid tumor. • Having diabetes mellitus. • History of or planned gastrectomy. • Having chronic lung disease (e.g., COPD, asthma, occupational or environmental lung disease). • Confirmed LTBI conversion within the past 2 years. • Healthcare worker. • Personnel working in congregate settings with a high risk of TB transmission (e.g., hospitals, schools) according to the TB Prevention Act. • Individuals considered at high risk for TB infection by local public health authorities (e.g., homeless persons, residents of shelters) under the TB Prevention Act.
	3. Individuals scheduled for LTBI testing, those who have already completed testing with confirmed results, or those diagnosed with LTBI who have initiated, are receiving, or have completed LTBI treatment within 2 years.
	4. Capable of understanding study procedures, voluntarily consenting to participate, and providing written informed consent.
Exclusion criteria	1. Aged < 19 years.
	2. History of treatment for active TB disease.
	3. Any condition deemed inappropriate for participation by the principal investigator.

COPD, chronic obstructive pulmonary disease; HIV, human immunodeficiency virus; LTBI, latent tuberculosis infection; TB, tuberculosis; TNF, tumor necrosis factor

During the study period, when a patient meeting the eligibility criteria is identified, the attending physician will notify the study coordinator or investigator. The investigator will review the patient’s eligibility according to the inclusion and exclusion criteria, provide a thorough explanation of the study objectives, procedures, potential risks, and personal data protection, and obtain written informed consent from those who voluntarily agree to participate. Eligible participants who have provided consent will then be formally enrolled in the study.

### Study population and analytic framework

**Fig 1:**
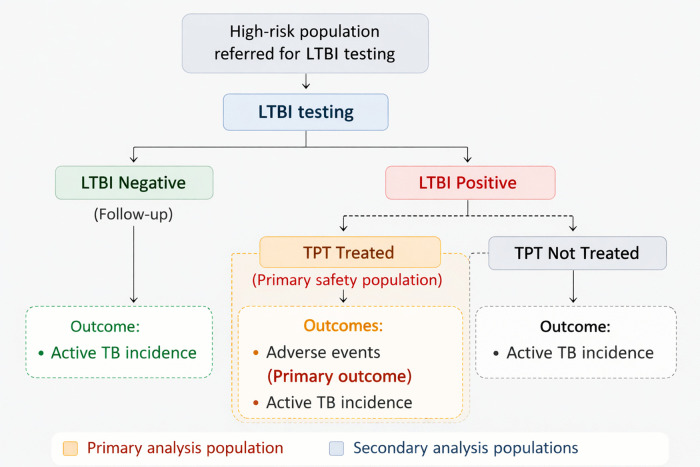
Study population and analysis framework of the STEP-TB cohort. LTBI, latent tuberculosis infection; TB, tuberculosis; TPT, TB preventive treatment.

After enrollment, all individuals referred for LTBI testing will constitute the source population of the cohort and will be described in terms of baseline characteristics (Table 2). Participants will then be followed according to their LTBI test results and subsequent treatment status (Fig 1). Individuals with negative LTBI test results will be followed to monitor progression to active TB. Among those with positive LTBI test results, participants will be categorized according to whether they receive TPT. Participants receiving TPT will be followed for both adverse events related to TPT and the development of active TB. Participants with positive test results who do not receive TPT will be followed to evaluate the incidence of active TB for comparative assessment.

**Table 2:** Study populations and outcomes in the cohort.

Study population	Description	Purpose of analysis	Outcomes assessed
All individuals referred for LTBI testing	High-risk population undergoing LTBI testing	Description of source population	Baseline characteristics
LTBI test negative	Participants with negative LTBI test results	Assessment of natural risk of TB among test-negative individuals	Incident active TB
LTBI test positive	Participants with positive LTBI test results	Population eligible for TPT	Stratified analyses according to TPT status
LTBI positive, TPT treated	Participants receiving tuberculosis preventive treatment	Primary safety analysis population	Adverse events related to TPT; Incident active TB
LTBI positive, TPT not treated	Participants with LTBI who do not receive TPT	Comparative assessment of TB risk	Incident active TB

LTBI, latent tuberculosis infection; TB, tuberculosis; TPT, tuberculosis preventive treatment

### Study procedures

All study procedures will be conducted as part of routine clinical care. LTBI testing will be performed using interferon-gamma release assays (IGRA), and patient management will follow the national TB guidelines according to test results.

The baseline evaluation aims to obtain a comprehensive understanding of each participant’s general health status and social background. Sociodemographic assessment will include economic status, education level, marital status, occupation, smoking and alcohol history. Clinical evaluation will cover current symptoms, medical history, comorbidities, current medications, and vital signs (e.g., blood pressure, heart rate, height, and weight). Laboratory assessment will include LTBI testing, complete blood count, blood chemistry, glycated hemoglobin, fasting glucose, HIV antibody, hepatitis B surface antigen and antibody, and hepatitis C antibody to evaluate baseline health and infection status.

To exclude active TB, additional history taking and diagnostic testing will be performed if necessary. Symptoms suggestive of active TB include persistent cough, sputum production, weight loss, and night sweats. When such symptoms are present, chest radiography will be performed as the initial evaluation. If radiographic findings are indeterminate, the attending physician may perform chest computed tomography for further clarification. Bacteriological testing will also be conducted, including acid-fast bacilli smear and culture, and nucleic acid amplification tests such as Xpert MTB/RIF assay, to confirm or rule out active TB infection.

Participants who initiate TPT will be systematically followed according to the national TB treatment guidelines. The schedule of visits is summarized in Table 3. Clinical evaluations will be performed on Day 14 (Visit 1) and Day 28 (Visit 2) after treatment initiation, and subsequently followed until the end of treatment. The frequency of interim visits during therapy will be determined at the discretion of the treating physician.

**Table 3:** Study visit schedule and assessments during tuberculosis preventive treatment.

Visit	V0	V1	V2	V3	V4	V5
Visit name	Screening	Baseline	2 weeks	1 month	EOT	EOS
Timing		D0	W1-3	M1-2	M3-10	M11-15
Informed consent	O					
Medical history	O	O	O	O	O	O
TB preventive treatment		O	O	O	O	
Chest imaging		O			O	O
Blood tests		O	O	O	O	
Adherence assessment			O	O	O	
Adverse event assessment			O	O	O	
Blood sampling	O				O	
VAMS sampling			O	O		
Questionnaire		O				

V, visit; D, day; W, Week; M, Month; EOT, End of Treatment; EOS, End of Study; TB, tuberculosis; VAMS, volumetric absorptive microsampling.

To assess the development of incident active TB, an additional follow-up visit will be conducted 12 months after TPT initiation (end of study).

To evaluate drug pharmacokinetics and pharmacogenetic profiles, blood samples will be collected using Volumetric Absorptive Microsampling (VAMS). VAMS collection will be performed at the planned visits (Week 2 and Week 4), precisely 3 hours (±5 minutes) after the participant takes the anti-TB medication. Participants will be instructed to take the medication at a consistent time in the morning before breakfast, and the exact time and date of drug administration and sampling will be recorded in the case report form.

For participants with negative LTBI results, or for those who test positive but do not receive TPT, follow-up assessments will be conducted at 6 months and 12 months after baseline, after which the study will conclude (Table 4). The occurrence of active TB will be evaluated throughout the follow-up period.

**Table 4:** Study visit schedule and assessments for participants with negative latent tuberculosis infection results or without tuberculosis preventive treatment.

Visit	V0	V1	V3	V4
Visit name	Screening	Baseline	6 months	EOS
Timing	−D14	D0	M3–10	M11–15
Informed consent	O			
Medical history	O	O	O	O
Chest imaging		O	O	O
Questionnaire		O		

V, visit; D, day; W, Week; M, Month; EOT, End of Treatment; EOS, End of Study

Study participation may be discontinued under specific circumstances. Participants who withdraw their informed consent will discontinue further study procedures. Investigator may also discontinue participant’s involvement if, in their clinical judgment, continued participation is deemed inappropriate due to medical, safety, or other relevant considerations. Participants who discontinue study participation will not be excluded from the analysis. Instead, all available data up to the point of discontinuation will be retained and reported where applicable.

### TB preventive treatment

Standard regimens for TPT include short-course rifamycin-based options such as 4 months of daily rifampin (4R) or 3 months of daily isoniazid plus rifampin (3HR) [4]. For individuals at increased risk of drug–drug interactions or adverse events, 9 months of daily isoniazid monotherapy (9H) may be considered as an alternative. If treatment with 9H must be discontinued due to hepatotoxicity or other contraindications, 6 months of isoniazid monotherapy (6H) can be used as a secondary option.

### Outcome measures

The primary outcome is adverse events leading to permanent discontinuation of any drugs in the TPT regimen. This outcome will be defined as any clinical or laboratory event occurring during TPT that results in permanent discontinuation of one or more drugs in the prescribed regimen. For example, in patients receiving a 3HR regimen, discontinuation of rifampicin due to adverse events while continuing isoniazid to complete TPT will be considered as meeting the primary outcome.

The secondary outcomes are completion of TPT, the cascade of care for TPT, and the preventive effectiveness of TPT. TPT completion will be defined as completion of the prescribed TPT regimen within the recommended treatment period according to national guidelines. TPT cascade of care will be described by evaluating the proportions of individuals who are eligible for TPT, initiate treatment, and successfully complete treatment. The preventive effectiveness of TPT will be evaluated the incidence of progression to active TB during the follow-up period.

### Adverse event assessment

Adverse events are defined as any undesirable and unintended signs, symptoms, or diseases temporally associated with the use of a medicinal product, regardless of causal relationship, including abnormal laboratory findings. The definition and severity grading of adverse events will follow the Common Terminology Criteria for Adverse Events (CTCAE), version 5.0.[6] At the time points specified in Table 2, clinical symptoms, blood laboratory tests, and chest radiography will be assessed. Adverse events will be recorded using the standardized adverse event reporting forms provided in the S1 Table. The reporting form captures the presence of adverse events, the reported event term coded using the MedDRA terminology, data of onset and resolution, seriousness, severity grade according to CTCAE, causality assessment with anti-TB drugs, action taken, and clinical outcome of the event. Adverse events will be evaluated in terms of their type, frequency, and severity. The clinical significance of each event will be determined by the investigator based on medical judgment and patient condition.

### Treatment adherence and completion

Treatment adherence will be assessed based on patient self-report during scheduled study visits by comparing the total number of prescribed treatment days with the number of days the medication was actually taken. If treatment is discontinued, the reason for discontinuation will be recorded. Treatment completion will be determined according to the type and duration of the TPT regimen. Completion of the 9H will be defined as taking at least 80% of the prescribed doses within 12 months. For the 4R, completion will be defined as taking at least 80% of the prescribed doses within 6 months. For the 3HR, completion will be defined as taking at least 80% of the prescribed doses within 4 months.

### Ascertainment of incident active TB during follow-up

The occurrence of active TB during the follow-up period will be assessed through scheduled study visits and telephone follow-up when participants are unable to attend in person. At follow-up visits, participants will undergo clinical evaluation including symptom screening and chest radiography. If active TB is suspected based on symptoms or radiographic findings, additional microbiological testing, including sputum smear microscopy, nucleic acid amplification testing, and mycobacterial culture, will be performed according to standard clinical practice. Incident active TB will be defined as microbiologically confirmed or clinically diagnosed TB occurring after study enrollment during the follow-up period.

### Statistical analysis

Baseline characteristics of participants undergoing LTBI testing will be summarized using means and standard deviations for normally distributed continuous variables, medians and interquartile ranges for non-normally distributed variables, and frequencies with percentages for categorical variables.

To investigate if the presence of CKD alters the risk of adverse events (present/absent) during TPT, logistic regression models will be built, initially categorizing CKD as a binary variable (present/absent) and then as a ternary variable (present severe, present and not severe, absent). The minimal sufficient adjustment set for inclusion in adjusted models will be derived from a directed acyclic graph (DAG). Potential effect modification by CKD treatment will be explored amongst individuals with CKD. All statistical analyses will be performed using R or SPSS software. Additional analyses will be considered further categorizing the outcome, e.g., on the basis of adverse event severity (ordinal logistic regression) or taking into account the association between an adverse event and the drugs in use. P-values will be corrected for multiple testing.

Assessment of the association between CKD and TPT completion will take place similarly, as will progression to active TB, albeit here using time-to-event models. Finally, the cascade of care analysis will be descriptive.

### Sample size calculation

CKD was defined according to the Kidney Disease: Improving Global Outcomes (KDIGO) guidelines as an estimated glomerular filtration rate (eGFR) <60 mL/min/1.73 m² for at least 3 months or evidence of kidney damage.[7] This study will evaluate whether CKD, a major medical high-risk condition for TB, is associated with adverse events during TPT. It is anticipated that approximately 40% of individuals undergoing LTBI testing will be LTBI-positive and initiate TPT, of whom about 15% will have CKD. Assuming adverse event rates of 20% in the CKD group and 10% in the non-CKD group, a total of 548 TPT initiators will be required to detect this difference with a two-sided α of 0.05 and 80% power.

Based on an expected LTBI positivity rate of 40%, approximately 1,370 LTBI testing candidates need to be enrolled to yield a sufficient number of treatment initiators. This sample size will provide adequate statistical power to assess whether CKD is an independent risk factor for adverse events during TPT. A total target enrollment of 1,370 participants is planned, with competitive enrollment implemented across participating sites; per-site recruitment targets may be adjusted accordingly.

### Ethics

This study will be conducted in accordance with the principles of the Declaration of Helsinki and the Good Clinical Practice guidelines. The study protocol has been reviewed and approved by the Institutional Review Board of Catholic Medical Center, The Catholic University of Korea (Approval number; XC25ENDI0053). This approval covers all participating sites in this multi-center study under a centralized institutional review board review process; therefore, no additional site-specific ethics approvals were required. Any major protocol amendments will be submitted for additional IRB approval prior to implementation.

Written informed consent will be obtained from all participants before enrollment. During the consent process, participants will receive detailed information regarding the study objectives, procedures, potential risks, and confidentiality safeguards. They will also be informed that participation is voluntary and that they may withdraw from the study at any time without any impact on their clinical care.

All participant data will be de-identified and managed using a secure, password-protected database. Only authorized research personnel will have access to identifiable information, and data handling will comply with the Personal Information Protection Act of the Republic of Korea. Data quality and consistency will be regularly monitored by the study coordination center.

### Dissemination

The study findings will be disseminated through peer-reviewed journals, national and international scientific conferences, and collaborative research networks. Summary results may also be shared with relevant public health authorities to inform national TB control policies. No personally identifiable information will be disclosed in any reports or publications

## Discussion

This prospective, multicenter cohort study conducted in a setting with an ongoing TB burden aims to generate robust evidence on the safety of TPT among individuals at high risk of developing active TB. The overarching goal of this study is to establish a well-characterized cohort of high-risk participants, accompanied by comprehensive clinical data and a biospecimen repository, to facilitate future translational and mechanistic research.

Accumulating evidence suggests that restricting TPT to currently recommended target groups is unlikely to be sufficient for TB elimination [8,9]. A large national analysis of the LTBI screening program in Korea [10] demonstrated that only a small fraction of individuals with positive IGRA results are captured by existing LTBI strategies, which primarily focus on close contacts of active TB cases and a limited number of medical high-risk conditions. Even when moderate-risk conditions such as diabetes mellitus were included, the proportion of the latent TB reservoir covered by current guidelines remained small. Moreover, a substantial burden of LTBI was observed outside the scope of conventional contact investigation and guideline-defined high-risk groups, particularly among older adults and individuals with multiple comorbidities. Together, these findings indicate that strategies confined to traditional risk categories are unlikely to sufficiently reduce the LTBI reservoir or achieve meaningful long-term declines in TB incidence [11]. In this context, expanding LTBI treatment eligibility, while ensuring safety through careful risk stratification, represents a critical component of TB elimination efforts in countries with an intermediate TB burden.

In addition to traditionally recognized high-risk conditions, this cohort includes participants with CKD (including those on dialysis), chronic respiratory disease (including those with occupational or environmental lung disease), and individuals receiving immunosuppressive therapy, biologic agents, or cancer treatment. By encompassing these populations, the STEP-TB cohort will provide stratified, evidence-based insights to guide more targeted and safer LTBI treatment strategies in high-risk groups.

CKD is an important high-risk condition that warrants inclusion in this study because it is consistently associated with a markedly increased risk of TB [12], largely driven by reactivation of latent infection rather than new exposure. As renal function declines, particularly in patients approaching or receiving dialysis, immune dysregulation substantially increases the likelihood of progression from LTBI to active TB disease, with reported TB incidence in dialysis populations far exceeding that of the general population [13]. In addition, TB in patients with CKD often presents atypically, leading to delayed diagnosis, poorer outcomes, and increased risk of transmission within dialysis units, thereby posing both individual and public health concerns [14]. Despite these risks, LTBI screening and treatment practices in CKD remain highly variable, largely due to diagnostic uncertainty and concerns about treatment-related adverse events, and robust, condition-specific safety data are lacking [15,16]. Recent reviews [17] have highlighted the urgent need for prospective evidence to better define the balance of benefit and harm of LTBI treatment in CKD populations and to support more consistent, evidence-based preventive strategies. By including patients with CKD across disease stages, the STEP-TB cohort is well positioned to address these critical evidence gaps and inform safer, more effective LTBI management in this high-risk group.

Importantly, this study is also designed to examine the cascade of care for LTBI [18,19], from diagnostic testing and treatment initiation to treatment completion, across different comorbidity groups. By systematically evaluating barriers and drop-offs at each step of the LTBI care continuum, STEP-TB will identify condition-specific hurdles faced by patients with complex medical comorbidities. Understanding these real-world challenges is essential for informing feasible and safe expansion of LTBI treatment policies, and the evidence generated by this cohort will directly support efforts to broaden preventive treatment strategies while maintaining patient safety.

In clinical practice, concerns about adverse events often lead clinicians to defer TB preventive treatment [20], even after LTBI is diagnosed, particularly in patients with complex comorbidities. This reflects a persistent lack of condition-specific safety data for LTBI treatment. While randomized controlled trials provide rigorous safety assessments, such as RIFAKiD-TB trial [21], their restricted eligibility and controlled settings may limit generalizability to real-world practice. As a prospective observational cohort, STEP-TB complements existing evidence by including diverse high-risk populations and capturing both anticipated and unanticipated adverse events under routine clinical care. The resulting real-world safety data can inform condition-specific risk assessment, support shared decision-making, and provide essential evidence for the safe expansion of LTBI preventive treatment strategies. The availability of condition-specific, real-world safety data may also enhance patient-centered care by enabling clearer, evidence-based communication about the risks and benefits of TPT for individuals undergoing or considered for LTBI testing.

In conlcusion, The STEP-TB study is a multicenter prospective observational cohort designed to generate real-world evidence on the safety and implementation of TPT in individuals at high risk of developing active TB. By systematically evaluating adverse events, treatment initiation, and completion across diverse high-risk populations, this study aims to inform clinical practice, patient-centered decision-making, and national TB control policies, thereby supporting the safe expansion of LTBI treatment strategies and contributing to TB elimination efforts in Korea.

## Supporting information

S1 Table Classification and severity assessment of adverse events during tuberculosis preventive treatment. (DOCX)
