# Supplementary material for: Study on the safety evaluation of latent tuberculosis treatment in high‑risk groups for tuberculosis development: Study protocol for a multi‑center prospective observational cohort study in Korea (STEP-TB)
Source: PLoS One. 2026 Jun 9;21(6):e0350186. doi: 10.1371/journal.pone.0350186 (PMC13249163; doi:10.1371/journal.pone.0350186)
Supplement: S1 Table — (DOCX) [file pone.0350186.s001.docx]

**S1 Table.** Classification and severity assessment of adverse events during tuberculosis preventive treatment.

| Field / Description | Options / Format |
| --- | --- |
| Presence of Adverse Event | 1: Yes 2: No |
| Adverse Event Term | Recorded using MedDRA terminology |
| Start Date of Adverse Event | YYYY-MM-DD |
| End Date of Adverse Event | YYYY-MM-DD |
| Serious Adverse Drug Reaction (SADR) | 1: Yes (Serious) 2: No (Not serious) |
| Severity | 1: Death 2: Life-threatening or disabling 3: Severe 4: Moderate 5: Mild |
|  | Severity will be graded according to CTCAE Version 6.0 |
| Causality Assessment with Anti-tuberculosis Drugs | 1: Definite 2: Probable 3: Possible 4: Doubtful (Unlikely) 5: Not related 6: Unable to evaluate |
|  | Cases assessed as 'Doubtful (Unlikely)' or 'Not related' will not be collected as adverse drug reaction data |
| Suspected Drug | 1: INH (Isoniazid) 2: RIF (Rifampicin) 3: INH + RIF |
| Action Taken for Adverse Drug Reaction | 1: No action taken 2: Medication treatment 3: Non-pharmacological treatment 4: Other |
| If 'Other', specify | Free text |
| Outcome of Adverse Drug Reaction | 1: Recovered 2: Recovering 3: Recovered with sequelae 4: Not recovered 5: Fatal 6: Unknown |
