# Supplementary material for: Study on the safety evaluation of latent tuberculosis treatment in high‑risk groups for tuberculosis development: Study protocol for a multi‑center prospective observational cohort study in Korea (STEP-TB)
Source: PLoS One. 2026 Jun 9;21(6):e0350186. doi: 10.1371/journal.pone.0350186 (PMC13249163; doi:10.1371/journal.pone.0350186)
Supplement: Attachment — Submitted filename: Response to reviewer.docx [file pone.0350186.s003.docx]

Editor office’s comment

**[Comment #0-1]** Please ensure that your manuscript meets PLOS ONE's style requirements, including those for file naming.

**[Response #0-1]** Thank you for your comment. We have reviewed the manuscript and ensured that it complies with PLOS ONE’s style requirements, including file naming conventions.

**[Comment #0-2]** Thank you for stating the following in the Acknowledgments Section of your manuscript:

“The STEP-TB cohort is supported by the Korea National Institute of Health (KNIH) through the Research Program on the “Study on the safety evaluation of latent tuberculosis treatment in high risk groups for tuberculosis development” (2025E200100). The funder has no role in the design of the study, data collection, analysis, interpretation, or the decision to submit the manuscript for publication.”

We note that you have provided funding information that is not currently declared in your Funding Statement. However, funding information should not appear in the Acknowledgments section or other areas of your manuscript. We will only publish funding information present in the Funding Statement section of the online submission form.

Please remove any funding-related text from the manuscript and let us know how you would like to update your Funding Statement. Currently, your Funding Statement reads as follows:

Authors receiving funding: JM, Grant number: 2025ER200600 Funder: Korea Disease Control and Prevention Agency (KDCA), Funder website: https://www.kdca.go.kr The funder had no role in the study design, data collection and analysis, decision to publish, or preparation of the manuscript.”

Please include your amended statements within your cover letter; we will change the online submission form on your behalf.

**[Response #0-2]** Thank you for your comment. We have removed all funding-related information from the Acknowledgments section. We would like to confirm the Funding Statement as follows:

“"This work was supported by the Research Program funded by the Korea Disease Control and Prevention Agency (2025ER200600). The funder had no role in study design, data collection and analysis, or preparation of the manuscript.”

**[Comment #0-3]** If the reviewer comments include a recommendation to cite specific previously published works, please review and evaluate these publications to determine whether they are relevant and should be cited. There is no requirement to cite these works unless the editor has indicated otherwise.

**[Response #0-3]** Thank you for your comment. The reviewers did not recommend any specific additional references; therefore, no changes were made regarding citations.

Reviewer #1: The protocol is well-written overall. However, I recommend elaborating on several items to ensure clarity and reproducibility:

**[Comment #1-1] Follow-up Procedures: Could you clarify the methodology for monitoring adverse events (AEs) and TB incidence during follow-up? Specifically:**

o Will you rely solely on symptom assessment or include routine laboratory tests?

o Will national TB registers (where available) be cross-referenced to ascertain cases?

**[Response 1-1]** Thank you for this helpful comment. We have clarified the follow-up procedures for monitoring AEs and TB incidence in the revised manuscript.

Adverse events are defined as any undesirable and unintended signs, symptoms, or diseases temporally associated with the use of a medicinal product, regardless of causal relationship, including abnormal laboratory findings. In this study, the definition and severity grading of AEs will follow the Common Terminology Criteria for Adverse Events (CTCAE), version 5.0. Participants with latent TB infection will receive standard TB preventive treatment according to current clinical guidelines. At the time points specified in Table 2, clinical symptoms, hematologic laboratory tests, and chest radiography will be assessed. Adverse events will be recorded using the adverse event reporting forms described in the Supplementary Materials.

“Adverse events are defined as any undesirable and unintended signs, symptoms, or diseases temporally associated with the use of a medicinal product, regardless of causal relationship, including abnormal laboratory findings. The definition and severity grading of adverse events will follow the Common Terminology Criteria for Adverse Events (CTCAE), version 5.0.[6] At the time points specified in **Table 2**, clinical symptoms, blood laboratory tests, and chest radiography will be assessed. Adverse events will be recorded using the standardized adverse event reporting forms provided in the **Supplementary Table 1**. The reporting form captures the presence of adverse events, the reported event term coded using the MedDRA terminology, data of onset and resolution, seriousness, severity grade according to CTCAE, causality assessment with anti-TB drugs, action taken, and clinical outcome of the event. Adverse events will be evaluated in terms of their type, frequency, and severity. The clinical significance of each event will be determined by the investigator based on medical judgment and patient condition.” (Methods, Page 12, Line 228-239)

Progression to active TB will be monitored through patient symptom assessment and chest radiography during follow-up.

“Ascertainment of incident active TB during follow-up

The occurrence of active TB during the follow-up period will be assessed through scheduled study visits and telephone follow-up when participants are unable to attend in person. At follow-up visits, participants will undergo clinical evaluation including symptom screening and chest radiography. If active TB is suspected based on symptoms or radiographic findings, additional microbiological testing, including sputum smear microscopy, nucleic acid amplification testing, and mycobacterial culture, will be performed according to standard clinical practice. Incident active TB will be defined as microbiologically confirmed or clinically diagnosed tuberculosis occurring after study enrollment during the follow-up period.” (Methods, Page 15, Line 272-280)

Cross-referencing with the national TB registry will not be performed for case ascertainment in this study.

**[Comment #1-2] Adverse Event Analysis: Regarding the regression analysis, which AEs will be evaluated?**

o Are you including all AEs regardless of severity, or focusing on Grade 3/4 events?

o Please specify if a standard grading system (e.g., CTCAE) will be used to assess severity.

**[Response #1-2]** Thank you for this helpful comment. We have clarified the adverse event monitoring and analysis procedures in the revised manuscript.

The primary outcome of this study is adverse events leading to permanent discontinuation of any drug in the TPT regimen, and these events will be included in the regression analysis.

“The primary outcome is adverse events leading to permanent discontinuation of any drugs in the TPT regimen. This outcome will be defined as any clinical or laboratory event occurring during TPT that results in permanent discontinuation of one or more drugs in the prescribed regimen. For example, in patients receiving a 3HR regimen, discontinuation of rifampicin due to adverse events while continuing isoniazid to complete TPT will be considered as meeting the primary outcome.” (Methods, Page 12, Line 216-220)

In addition, all adverse events occurring during TPT will be collected and descriptively analyzed in terms of their type, frequency, and severity. Severity will be graded according to the Common Terminology Criteria for Adverse Events (CTCAE), version 5.0.

“Adverse events are defined as any undesirable and unintended signs, symptoms, or diseases temporally associated with the use of a medicinal product, regardless of causal relationship, including abnormal laboratory findings. The definition and severity grading of adverse events will follow the Common Terminology Criteria for Adverse Events (CTCAE), version 5.0.[6] At the time points specified in **Table 2**, clinical symptoms, blood laboratory tests, and chest radiography will be assessed. Adverse events will be recorded using the standardized adverse event reporting forms provided in the **Supplementary Table 1**. The reporting form captures the presence of adverse events, the reported event term coded using the MedDRA terminology, data of onset and resolution, seriousness, severity grade according to CTCAE, causality assessment with anti-TB drugs, action taken, and clinical outcome of the event. Adverse events will be evaluated in terms of their type, frequency, and severity. The clinical significance of each event will be determined by the investigator based on medical judgment and patient condition.” (Methods, Page 12, Line 228-239)

**[Comment #1-3] Adherence and Completion: How will treatment adherence be measured (e.g., pill counts, self-reports, or pharmacy records), and what is your specific clinical definition of "treatment completion"?**

[Response #1-3] Thank you for the comment. We have clarified that treatment adherence will be assessed based on patient self-report and have added regimen-specific definitions of treatment completion in the Methods section.

“Treatment adherence and completion

Treatment adherence will be assessed based on patient self-report during scheduled study visits by comparing the total number of prescribed treatment days with the number of days the medication was actually taken. If treatment is discontinued, the reason for discontinuation will be recorded. Treatment completion will be determined according to the type and duration of the TPT regimen. Completion of the 9H will be defined as taking at least 80% of the prescribed doses within 12 months. For the 4R, completion will be defined as taking at least 80% of the prescribed doses within 6 months. For the 3HR, completion will be defined as taking at least 80% of the prescribed doses within 4 months.” (Methods, Page 13, Line 243-251)

**[Comment #1-4] Definition of CKD: Please provide the specific diagnostic criteria or staging used to define Chronic Kidney Disease (CKD) in this study.**

**[Response #1-4]** Thank you for this helpful comment. We have clarified the definition of chronic kidney disease (CKD) in the Methods section(simple size calculation). CKD was defined according to the Kidney Disease: Improving Global Outcomes (KDIGO) guidelines based on the estimated glomerular filtration rate (eGFR).

“CKD was defined according to the Kidney Disease: Improving Global Outcomes (KDIGO) guidelines as an estimated glomerular filtration rate (eGFR) <60 mL/min/1.73 m² for at least 3 months or evidence of kidney damage.” (Methods, Page 15, Line 283-285)

**[Comment #1-5] Structural Flow: The fact that CKD is the primary exposure variable only becomes clear in the analysis section. I recommend flagging this—along with its scientific rationale—much earlier in the Introduction.**

[Response #1-6] Thank you for this helpful comment. We agree that the role of chronic kidney disease (CKD) as an important exposure variable should be clarified earlier in the manuscript. Therefore, we have revised the Introduction to explicitly highlight CKD as a key high-risk condition and to explain the scientific rationale for evaluating the association between CKD and adverse events during tuberculosis preventive treatment.

“Among the medical high-risk groups for TB, including individuals with chronic kidney disease (CKD), chronic lung disease, diabetes mellitus, malignancy, immunosuppressive conditions, and occupational exposure, the safety of tuberculosis preventive treatment remains an important clinical concern. CKD is of special interest because patients with CKD have an increased risk of TB and may be more vulnerable to drug-related adverse events. This study aims to evaluate the safety of TPT in individuals at high risk of developing active TB disease in Korea using a multicenter prospective cohort design. The primary objective is to specifically investigate whether CKD is associated with an increased risk of adverse events during TPT. The secondary objective is to assess the association between CKD and TPT completion, to describe the TPT cascade of care among individuals at high risk of TB, and to evaluate the preventive effectiveness of TPT in reducing progression to active TB during the follow-up period. These objectives will allow a comprehensive understanding of both the safety and effectiveness of TPT in high-risk populations.” (Introduction, Page 5, Line 87-95.)

**[Comment #1-6] Submission Requirements: I have reviewed the PLOS submission guidelines; it remains unclear whether a formal Discussion section is mandatory for protocol manuscripts. I am happy to defer to the Editor’s guidance on this.**

[Response #1-6] Thank you for this comment. A brief Discussion section has been included in the manuscript to summarize the expected contribution and implications of the STEP-TB cohort. We will defer to the Editor’s guidance regarding whether this section should be retained in the final version.

Reviewer #2: PONE 25-67189 Review

Interesting and valuable study, that is already funded and ongoing. As such hard to know how much can be changed. Suggestions to add for clarity of paper, if done. If not done authors can consider adding to study procedures, if feasible.

**[Comment #2-1] Objectives. The paper would be clearer if there was a clear statement of objectives at the end of the Intro, or start of methods. It appears the primary objective, based on statements made in the sample size section, is to compare the rate of adverse events in persons with CKD, but this is not stated anywhere until near the end of the methods section. The title mentions ‘safety evaluation in high risk persons’ but does not specify CKD. Hence, consistency and clarity is needed**

[Response #2-1] Thank you for this helpful comment. We agree that the study objectives should be stated more clearly in the manuscript. Accordingly, we have revised the Introduction to explicitly state the primary objective of the study at the end of the Introduction, as follows.

“Among the medical high-risk groups for TB, including individuals with chronic kidney disease (CKD), chronic lung disease, diabetes mellitus, malignancy, immunosuppressive conditions, and occupational exposure, the safety of tuberculosis preventive treatment remains an important clinical concern. CKD is of special interest because patients with CKD have an increased risk of TB and may be more vulnerable to drug-related adverse events. This study aims to evaluate the safety of TPT in individuals at high risk of developing active TB disease in Korea using a multicenter prospective cohort design. The primary objective is to specifically investigate whether CKD is associated with an increased risk of adverse events during TPT. The secondary objective is to assess the association between CKD and TPT completion, to describe the TPT cascade of care among individuals at high risk of TB, and to evaluate the preventive effectiveness of TPT in reducing progression to active TB during the follow-up period. These objectives will allow a comprehensive understanding of both the safety and effectiveness of TPT in high-risk populations.” (Introduction, Page 5, Line 87-95.)

Accordingly, the Outcome Measures section has also been updated to reflect these changes.

“The primary outcome is adverse events leading to permanent discontinuation of any drugs in the TPT regimen. This outcome will be defined as any clinical or laboratory event occurring during TPT that results in permanent discontinuation of one or more drugs in the prescribed regimen. For example, in patients receiving a 3HR regimen, discontinuation of rifampicin due to adverse events while continuing isoniazid to complete TPT will be considered as meeting the primary outcome.

The secondary outcome is completion of TPT, the cascade of care for TPT and the preventive effectiveness of TPT. This will be defined as completion of the prescribed TPT regimen within the recommended treatment period according to national guidelines. TPT cascade of care will be described by evaluating the proportions of individuals who are eligible for TPT, initiate treatment, and successfully complete treatment. The preventive effectiveness of TPT will be evaluated the incidence of progression to active TB during the follow-up period.” (Methods, Page 12, Line 224-235)

We have also clarified that the study evaluates the safety of tuberculosis preventive treatment in individuals at high risk for TB, while specifically examining whether CKD is associated with an increased risk of adverse events during treatment. These revisions improve the clarity and consistency between the study rationale, objectives, and analysis plan in the revised manuscript.

**[Comment #2-2] Population. The primary objective is safety, so one expects the study population will be those treated. At times (Line 81) the study population is all those with a positive TB infection test. But in other places – the TBI test negative persons, and the Test positive but not treated – are also studied. So a listing summarizing the various study populations to be included, and for what objectives and analyses would also be helpful. As well, some sense of the larger population from those referred for TBI testing is drawn would be useful, for later generalizing of results.**

**[Response #2-2]** Thank you for this helpful comment. We agree that the description of the study population could be clarified. The STEP-TB cohort includes individuals undergoing LTBI testing in high-risk populations. Participants are followed according to their LTBI status and treatment status. Individuals with negative LTBI test results are followed to monitor progression to active TB. Among those with positive LTBI test results, participants are categorized according to whether they receive TPT. Participants receiving TPT are evaluated for both adverse events and progression to active TB, whereas those not receiving TPT are followed for TB incidence.

To further clarify the study population structure and the corresponding analyses, we have revised the Methods section and added a schematic flow diagram illustrating the study populations according to LTBI status and treatment status in the revised manuscript (Table 2 and Figure 1).

“Study population and analytic framework

After enrollment, all individuals referred for LTBI testing will constitute the source population of the cohort and will be described in terms of baseline characteristics (Table 2). Participants will then be followed according to their LTBI test results and subsequent treatment status (Figure 1). Individuals with negative LTBI test results will be followed to monitor progression to active TB. Among those with positive LTBI test results, participants will be categorized according to whether they receive TPT. Participants receiving TPT will be followed for both adverse events related to TPT and the development of active TB. Participants with positive test results who do not receive TPT will be followed to evaluate the incidence of active TB for comparative assessment.“ (Methods, Page 8, Line 138-146)

Table 2. Study populations and outcomes in the cohort

| Study population | Description | Purpose of analysis | Outcomes assessed |
| --- | --- | --- | --- |
| All individuals referred for LTBI testing | High-risk population undergoing LTBI testing | Description of source population | Baseline characteristics |
| LTBI test negative | Participants with negative LTBI test results | Assessment of natural risk of TB among test-negative individuals | Incident active TB |
| LTBI test positive | Participants with positive LTBI test results | Population eligible for TPT | Stratified analyses according to TPT status |
| LTBI positive, TPT treated | Participants receiving tuberculosis preventive treatment | Primary safety analysis population | Adverse events related to TPT; Incident active TB |
| LTBI positive, TPT not treated | Participants with LTBI who do not receive TPT | Comparative assessment of TB risk | Incident active TB |

LTBI, latent tuberculosis infection; TB, tuberculosis; TPT, tuberculosis preventive treatment

Figure 1. Study population and analysis framework of the STEP-TB cohort

LTBI, latent tuberculosis infection; TB, tuberculosis; TPT, TB preventive treatment

**[Comment #2-3] Cascade analysis. Not stated, but should be possible with the design is a cascade analysis from ‘contemplating TB infection testing’ to completed TPT. This would be interesting, especially if they can compare characteristics of those who are lost at each step (not tested vs tested, test negative vs test positive, not treated vs start TPT, not completed vs completed TPT).**

[Response #2-3] Thank you for this valuable suggestion. The STEP-TB cohort design allows assessment of different stages from LTBI testing to TPT. We agree that cascade analyses provide useful insights into gaps in LTBI testing and treatment in high-risk populations. Such analyses may be explored in future analyses using the STEP-TB cohort dataset.

“The secondary objective is to assess the association between CKD and TPT completion, to describe the TPT cascade of care among individuals at high risk of TB, and to evaluate the preventive effectiveness of TPT in reducing progression to active TB during the follow-up period.” [Introduction, Page 5, Line 94-97]

“TPT cascade of care will be described by evaluating the proportions of individuals who are eligible for TPT, initiate treatment, and successfully complete treatment.” [Methods, Page 12, Line 222-224]

**[Comment #2-4] Procedures and outcome definitions: Completion of TPT – how will that be measured, and when. How will completion be defined? Same for Adverse events – how will they be detected, investigated and then defined. Grading? Using what grading system? Serious? Drug discontinuation? What about type of AE, and relationship to TPT regimen?**

[Response #2-4] Thank you for this helpful comment. We have clarified the procedures for assessing treatment completion and adverse events in the Methods section. Treatment adherence will be assessed based on patient self-report during scheduled study visits, and treatment completion is defined using regimen-specific criteria (≥80% of prescribed doses within the recommended treatment period for 9H, 4R, and 3HR).

We have also expanded the description of adverse event monitoring. Adverse events will be detected during scheduled study visits and recorded using standardized adverse event reporting forms. The type, frequency, and severity of adverse events will be documented. Severity will be graded according to the CTCAE), version 5.0. Information on seriousness, suspected drug, action taken (including drug discontinuation), and causality with the TPT regimen will also be recorded.

“Adverse event assessment

Adverse events are defined as any undesirable and unintended signs, symptoms, or diseases temporally associated with the use of a medicinal product, regardless of causal relationship, including abnormal laboratory findings. The definition and severity grading of adverse events will follow the Common Terminology Criteria for Adverse Events (CTCAE), version 5.0.[6] At the time points specified in Table 2, clinical symptoms, blood laboratory tests, and chest radiography will be assessed. Adverse events will be recorded using the standardized adverse event reporting forms provided in the Supplementary Table 1. The reporting form captures the presence of adverse events, the reported event term coded using the MedDRA terminology, data of onset and resolution, seriousness, severity grade according to CTCAE, causality assessment with anti-TB drugs, action taken, and clinical outcome of the event. Adverse events will be evaluated in terms of their type, frequency, and severity. The clinical significance of each event will be determined by the investigator based on medical judgment and patient condition.” [Methods, Page 12, Line 229-241]

“Treatment adherence and completion

Treatment adherence will be assessed based on patient self-report during scheduled study visits by comparing the total number of prescribed treatment days with the number of days the medication was actually taken. If treatment is discontinued, the reason for discontinuation will be recorded. Treatment completion will be determined according to the type and duration of the TPT regimen. Completion of the 9H will be defined as taking at least 80% of the prescribed doses within 12 months. For the 4R, completion will be defined as taking at least 80% of the prescribed doses within 6 months. For the 3HR, completion will be defined as taking at least 80% of the prescribed doses within 4 months.” [Methods, Page 13, Line 243-251]

**[Comment #2-5] Terminology – a minor point, but WHO now recommends thew terms: TB infection (instead of “latent TB infection” – introduced by CDC/ATS in 2000), and TB disease (instead of ‘active TB’). For consideration.**

[Response #2-5] Thank you for this helpful suggestion. We acknowledge that the WHO recommends the terms “TB infection” and “TB disease.” However, as the terminology “latent tuberculosis infection (LTBI)” is still widely used in clinical guidelines and research literature, including the Korean Guidelines for Tuberculosis, we have retained the term LTBI in this manuscript for consistency.

**[Comment #2-6] Excluding participants. Line 181 – they suggest that persons who are lost to Follow-up will be excluded. But, this is an outcome, and such participants should not be excluded.**

[Response #2-6] Thank you for this important comment. We agree that participants lost to follow-up should not be excluded from the analysis. Accordingly, we have revised the manuscript to clarify that although study participation may be terminated in cases such as loss to follow-up, these participants will not be excluded from the analysis and their available data will be included where applicable.

“Study participation may be discontinued under specific circumstances. Participants who withdraw their informed consent will discontinue further study procedures. Investigator may also discontinue participant’s involvement if, in their clinical judgment, continued participation is deemed inappropriate due to medical, safety, or other relevant considerations. Participants who discontinue study participation will not be excluded from the analysis. Instead, all available data up to the point of discontinuation will be retained and reported where applicable.” (Methods, Page 12, Line 207-212)
