# Supplementary material for: Study on the safety evaluation of latent tuberculosis treatment in high‑risk groups for tuberculosis development: Study protocol for a multi‑center prospective observational cohort study in Korea (STEP-TB)
Source: PLoS One. 2026 Jun 9;21(6):e0350186. doi: 10.1371/journal.pone.0350186 (PMC13249163; doi:10.1371/journal.pone.0350186)
Supplement: Peer review history

# Peer review: Decision Letter 0

**DOI:** 10.1371/journal.pone.0350186.r001

**Authors:** Violet Chihota

17 Feb 2026

PONE-D-25-67189

Study on The Safety Evaluation of Latent Tuberculosis Treatment in High‑Risk Groups for Tuberculosis Development : Study Protocol for a Multi‑center Prospective Observational Cohort Study in Korea (STEP-TB)

PLOS One

Dear Dr. Min,

Thank you for submitting your manuscript to PLOS ONE. After careful consideration, we feel that it has merit but does not fully meet PLOS ONE’s publication criteria as it currently stands. Therefore, we invite you to submit a revised version of the manuscript that addresses the points raised during the review process.

Please submit your revised manuscript by Apr 03 2026 11:59PM. If you will need more time than this to complete your revisions, please reply to this message or contact the journal office at plosone@plos.org. When you're ready to submit your revision, log on to https://www.editorialmanager.com/pone/ and select the 'Submissions Needing Revision' folder to locate your manuscript file.

Please include the following items when submitting your revised manuscript:

- A letter that responds to each point raised by the academic editor and reviewer(s). You should upload this letter as a separate file labeled 'Response to Reviewers'.
- A marked-up copy of your manuscript that highlights changes made to the original version. You should upload this as a separate file labeled 'Revised Manuscript with Track Changes'.
- An unmarked version of your revised paper without tracked changes. You should upload this as a separate file labeled 'Manuscript'.

If you would like to make changes to your financial disclosure, please include your updated statement in your cover letter. Guidelines for resubmitting your figure files are available below the reviewer comments at the end of this letter.

If applicable, we recommend that you deposit your laboratory protocols in protocols.io to enhance the reproducibility of your results. Protocols.io assigns your protocol its own identifier (DOI) so that it can be cited independently in the future. For instructions see: https://journals.plos.org/plosone/s/submission-guidelines#loc-laboratory-protocols. Additionally, PLOS ONE offers an option for publishing peer-reviewed Lab Protocol articles, which describe protocols hosted on protocols.io. Read more information on sharing protocols at https://plos.org/protocols?utm_medium=editorial-email&utm_source=authorletters&utm_campaign=protocols.

We look forward to receiving your revised manuscript.

Kind regards,

Violet Chihota

Academic Editor

PLOS One

Journal Requirements:

When submitting your revision, we need you to address these additional requirements.

1. Please ensure that your manuscript meets PLOS ONE's style requirements, including those for file naming. The PLOS ONE style templates can be found at

https://journals.plos.org/plosone/s/file?id=wjVg/PLOSOne_formatting_sample_main_body.pdf and

https://journals.plos.org/plosone/s/file?id=ba62/PLOSOne_formatting_sample_title_authors_affiliations.pdf

2. Thank you for stating the following in the Acknowledgments Section of your manuscript:

“The STEP-TB cohort is supported by the Korea National Institute of Health (KNIH) through the Research Program on the “Study on the safety evaluation of latent tuberculosis treatment in high risk groups for tuberculosis development” (2025E200100). The funder has no role in the design of the study, data collection, analysis, interpretation, or the decision to submit the manuscript for publication.”

We note that you have provided funding information that is not currently declared in your Funding Statement. However, funding information should not appear in the Acknowledgments section or other areas of your manuscript. We will only publish funding information present in the Funding Statement section of the online submission form.

Please remove any funding-related text from the manuscript and let us know how you would like to update your Funding Statement. Currently, your Funding Statement reads as follows:

Authors receiving funding: JM, Grant number: 2025ER200600 Funder: Korea Disease Control and Prevention Agency (KDCA), Funder website: https://www.kdca.go.kr The funder had no role in the study design, data collection and analysis, decision to publish, or preparation of the manuscript.”

Please include your amended statements within your cover letter; we will change the online submission form on your behalf.

3. If the reviewer comments include a recommendation to cite specific previously published works, please review and evaluate these publications to determine whether they are relevant and should be cited. There is no requirement to cite these works unless the editor has indicated otherwise.

[Note: HTML markup is below. Please do not edit.]

Reviewers' comments:

Reviewer's Responses to Questions

**Comments to the Author**

1. Does the manuscript provide a valid rationale for the proposed study, with clearly identified and justified research questions?

The research question outlined is expected to address a valid academic problem or topic and contribute to the base of knowledge in the field.

Reviewer #1: Yes

Reviewer #2: Yes

**********

2. Is the protocol technically sound and planned in a manner that will lead to a meaningful outcome and allow testing the stated hypotheses?

The manuscript should describe the methods in sufficient detail to prevent undisclosed flexibility in the experimental procedure or analysis pipeline, including sufficient outcome-neutral conditions (e.g. necessary controls, absence of floor or ceiling effects) to test the proposed hypotheses and a statistical power analysis where applicable. As there may be aspects of the methodology and analysis which can only be refined once the work is undertaken, authors should outline potential assumptions and explicitly describe what aspects of the proposed analyses, if any, are exploratory.

Reviewer #1: Yes

Reviewer #2: Partly

**********

3. Is the methodology feasible and described in sufficient detail to allow the work to be replicable?

Descriptions of methods and materials in the protocol should be reported in sufficient detail for another researcher to reproduce all experiments and analyses. The protocol should describe the appropriate controls, sample size calculations, and replication needed to ensure that the data are robust and reproducible.

Reviewer #1: Yes

Reviewer #2: Yes

**********

4. Have the authors described where all data underlying the findings will be made available when the study is complete?

The PLOS Data policy requires authors to make all data underlying the findings described in their manuscript fully available without restriction, with rare exception, at the time of publication. The data should be provided as part of the manuscript or its supporting information, or deposited to a public repository. For example, in addition to summary statistics, the data points behind means, medians and variance measures should be available. If there are restrictions on publicly sharing data—e.g. participant privacy or use of data from a third party—those must be specified.

Reviewer #1: Yes

Reviewer #2: No

**********

5. Is the manuscript presented in an intelligible fashion and written in standard English?

PLOS ONE does not copyedit accepted manuscripts, so the language in submitted articles must be clear, correct, and unambiguous. Any typographical or grammatical errors should be corrected at revision, so please note any specific errors here.

Reviewer #1: Yes

Reviewer #2: Yes

**********

6. Review Comments to the Author

Please use the space provided to explain your answers to the questions above and, if applicable, provide comments about issues authors must address before this protocol can be accepted for publication. You may also include additional comments for the author, including concerns about research or publication ethics.

You may also provide optional suggestions and comments to authors that they might find helpful in planning their study.

(Please upload your review as an attachment if it exceeds 20,000 characters)

Reviewer #1: The protocol is well-written overall. However, I recommend elaborating on several items to ensure clarity and reproducibility:

1. Follow-up Procedures: Could you clarify the methodology for monitoring adverse events (AEs) and TB incidence during follow-up? Specifically:

o Will you rely solely on symptom assessment or include routine laboratory tests?

o Will national TB registers (where available) be cross-referenced to ascertain cases?

2. Adverse Event Analysis: Regarding the regression analysis, which AEs will be evaluated?

o Are you including all AEs regardless of severity, or focusing on Grade 3/4 events?

o Please specify if a standard grading system (e.g., CTCAE) will be used to assess severity.

3. Adherence and Completion: How will treatment adherence be measured (e.g., pill counts, self-reports, or pharmacy records), and what is your specific clinical definition of "treatment completion"?

4. Definition of CKD: Please provide the specific diagnostic criteria or staging used to define Chronic Kidney Disease (CKD) in this study.

5. Structural Flow: The fact that CKD is the primary exposure variable only becomes clear in the analysis section. I recommend flagging this—along with its scientific rationale—much earlier in the Introduction.

6. Submission Requirements: I have reviewed the PLOS submission guidelines; it remains unclear whether a formal Discussion section is mandatory for protocol manuscripts. I am happy to defer to the Editor’s guidance on this.

Reviewer #2: PONE 25-67189 Review

Interesting and valuable study, that is already funded and ongoing. As such hard to know how much can be changed. Suggestions to add for clarity of paper, if done. If not done authors can consider adding to study procedures, if feasible.

1. Objectives. The paper would be clearer if there was a clear statement of objectives at the end of the Intro, or start of methods. It appears the primary objective, based on statements made in the sample size section, is to compare the rate of adverse events in persons with CKD, but this is not stated anywhere until near the end of the methods section. The title mentions ‘safety evaluation in high risk persons’ but does not specify CKD. Hence, consistency and clarity is needed

2. Population. The primary objective is safety, so one expects the study population will be those treated. At times (Line 81) the study population is all those with a positive TB infection test. But in other places – the TBI test negative persons, and the Test positive but not treated – are also studied. So a listing summarizing the various study populations to be included, and for what objectives and analyses would also be helpful. As well, some sense of the larger population from those referred for TBI testing is drawn would be useful, for later generalizing of results.

3. Cascade analysis. Not stated, but should be possible with the design is a cascade analysis from ‘contemplating TB infection testing’ to completed TPT. This would be interesting, especially if they can compare characteristics of those who are lost at each step (not tested vs tested, test negative vs test positive, not treated vs start TPT, not completed vs completed TPT).

4. Procedures and outcome definitions: Completion of TPT – how will that be measured, and when. How will completion be defined? Same for Adverse events – how will they be detected, investigated and then defined. Grading? Using what grading system? Serious? Drug discontinuation? What about type of AE, and relationship to TPT regimen?

5. Terminology – a minor point, but WHO now recommends thew terms: TB infection (instead of “latent TB infection” – introduced by CDC/ATS in 2000), and TB disease (instead of ‘active TB’). For consideration.

6. Excluding participants. Line 181 – they suggest that persons who are lost to Follow-up will be excluded. But, this is an outcome, and such participants should not be excluded.

**********

7. PLOS authors have the option to publish the peer review history of their article (what does this mean?). If published, this will include your full peer review and any attached files.

If you choose “no”, your identity will remain anonymous but your review may still be made public.

**Do you want your identity to be public for this peer review?** For information about this choice, including consent withdrawal, please see our Privacy Policy.

Reviewer #1: **Yes:** Yohhei Hamada

Reviewer #2: **Yes:** dick Menzies

**********

[NOTE: If reviewer comments were submitted as an attachment file, they will be attached to this email and accessible via the submission site. Please log into your account, locate the manuscript record, and check for the action link "View Attachments". If this link does not appear, there are no attachment files.]

To ensure your figures meet our technical requirements, please review our figure guidelines: https://journals.plos.org/plosone/s/figures

You may also use PLOS’s free figure tool, NAAS, to help you prepare publication quality figures: https://journals.plos.org/plosone/s/figures#loc-tools-for-figure-preparation.

NAAS will assess whether your figures meet our technical requirements by comparing each figure against our figure specifications.

# Author comment: Author response to Decision Letter 1

**DOI:** 10.1371/journal.pone.0350186.r002

2 Apr 2026

We thank the reviewers and editors for their careful evaluation and constructive comments.

All comments have been addressed in the revised manuscript. Specifically, we have clarified the study objectives and highlighted the role of chronic kidney disease (CKD) as a key exposure variable in the Introduction. The Methods section has been revised to provide more detailed descriptions of the study population, follow-up procedures, treatment adherence and completion, and adverse event assessment using standardized criteria. A schematic flow diagram has been added to improve clarity of the study population and analysis framework.

We have also clarified that participants lost to follow-up will not be excluded from the analysis. In addition, terminology and outcome definitions have been revised for consistency, and all requested editorial changes, including funding information and ethical documentation, have been incorporated.

A detailed, point-by-point response to all reviewer comments is provided in the uploaded “Response to Reviewers” file.

Attachment Submitted filename: Response to reviewer.docx

# Editor report: Decision Letter 1

**DOI:** 10.1371/journal.pone.0350186.r003

**Authors:** Violet Chihota

11 May 2026

Study on The Safety Evaluation of Latent Tuberculosis Treatment in High‑Risk Groups for Tuberculosis Development: Study Protocol for a Multi‑center Prospective Observational Cohort Study in Korea (STEP-TB)

PONE-D-25-67189R1

Dear Dr. Jinsoo Min,

We’re pleased to inform you that your manuscript has been judged scientifically suitable for publication and will be formally accepted for publication once it meets all outstanding technical requirements.

Within one week, you’ll receive an e-mail detailing the required amendments. When these have been addressed, you’ll receive a formal acceptance letter and your manuscript will be scheduled for publication.

An invoice will be generated when your article is formally accepted. Please note, if your institution has a publishing partnership with PLOS and your article meets the relevant criteria, all or part of your publication costs will be covered. Please make sure your user information is up-to-date by logging into Editorial Manager at Editorial Manager® and clicking the ‘Update My Information' link at the top of the page. For questions related to billing, please contact billing support.

If your institution or institutions have a press office, please notify them about your upcoming paper to help maximize its impact. If they’ll be preparing press materials, please inform our press team as soon as possible -- no later than 48 hours after receiving the formal acceptance. Your manuscript will remain under strict press embargo until 2 pm Eastern Time on the date of publication. For more information, please contact onepress@plos.org.

Kind regards,

Violet Chihota

Academic Editor

PLOS One

Additional Editor Comments (optional):

Reviewers' comments:

# Editor report: Acceptance letter

**DOI:** 10.1371/journal.pone.0350186.r004

**Authors:** Violet Chihota

PONE-D-25-67189R1

PLOS One

Dear Dr. Min,

I'm pleased to inform you that your manuscript has been deemed suitable for publication in PLOS One. Congratulations! Your manuscript is now being handed over to our production team.

At this stage, our production department will prepare your paper for publication. This includes ensuring the following:

* All references, tables, and figures are properly cited

* All relevant supporting information is included in the manuscript submission,

* There are no issues that prevent the paper from being properly typeset

You will receive further instructions from the production team, including instructions on how to review your proof when it is ready. Please keep in mind that we are working through a large volume of accepted articles, so please give us a few days to review your paper and let you know the next and final steps.

Lastly, if your institution or institutions have a press office, please let them know about your upcoming paper now to help maximize its impact. If they'll be preparing press materials, please inform our press team within the next 48 hours. Your manuscript will remain under strict press embargo until 2 pm Eastern Time on the date of publication. For more information, please contact onepress@plos.org.

You will receive an invoice from PLOS for your publication fee after your manuscript has reached the completed accept phase. If you receive an email requesting payment before acceptance or for any other service, this may be a phishing scheme. Learn how to identify phishing emails and protect your accounts at https://explore.plos.org/phishing.

If we can help with anything else, please email us at customercare@plos.org.

Thank you for submitting your work to PLOS ONE and supporting open access.

Kind regards,

PLOS ONE Editorial Office Staff

on behalf of

Dr. Violet Chihota

Academic Editor

PLOS One
